# Anti-Wrinkle Benefits of Peptides Complex Stimulating Skin Basement Membrane Proteins Expression

**DOI:** 10.3390/ijms21010073

**Published:** 2019-12-20

**Authors:** Sekyoo Jeong, Seokjeong Yoon, Sungwoo Kim, Juyeon Jung, Myungho Kor, Kayoung Shin, Chaejin Lim, Hyo Sun Han, Haekwang Lee, Kyeong-Yong Park, Jinwan Kim, Hwa Jee Chung, Hyun Jung Kim

**Affiliations:** 1 Research Division, Incospharm Corporation, Daejeon 34036, Korea; sekyoo.jeong@incospharm.com (S.J.); sjyoon@incospharm.com (S.Y.); swkim@incospharm.com (S.K.); jjy6519@incospharm.com (J.J.); mhkor@incospharm.com (M.K.); kayoung0671@incospharm.com (K.S.); cjlim@incospharm.com (C.L.); hjchung@incospharm.com (H.J.C.); 2 P&K Skin Research Center Company Limited, Seoul 07236, Korea; hs.han@pnkskin.com (H.S.H.); hklee@pnkskin.com (H.L.); 3 Research Division, Cha Meditech Corporation, Daejeon 34025, Korea; pky@chamc.co.kr; 4 CHA Bio F&C, Seongnam 13488, Korea; justinwkim@chamc.co.kr; 5 Department of Dermatology, CHA Bundang Medical Center, CHA University, Seongnam 13496, Korea

**Keywords:** dermal-epidermal junction, nidogen, laminin, wrinkle, clinical efficacy, peptides complex

## Abstract

The dermal-epidermal junction (DEJ) provides a physical and biological interface between the epidermis and the dermis. In addition to providing a structural integrity, the DEJ also acts as a passageway for molecular transport. Based on the recently reported importance of the DEJ in skin aging, novel peptide derivatives have been tested for their effects on basement membrane (BM) protein expressions in cultured human epidermal keratinocytes. As a result, protein expressions of collagen XVII, laminin and nidogen were stimulated by the test peptide and peptides complex. Further ex vivo evaluation using excised human skin, confirmed that the topical application of the peptides complex significantly increased dermal collagen expression, as well as expressions of collagen XVII and laminin. Interestingly, while the origin of the laminin protein is epidermal keratinocytes, the immunohistochemical staining of skin showed that laminin was only detected in the uppermost layer of the dermis, which suggests a tight assembly of laminin protein onto the dermal side of the DEJ. These results suggest that a peptide complex could improve the structural properties of the DEJ through its ability to stimulate BM proteins. In order to evaluate the anti-wrinkle benefits of the peptide complex in vivo, a clinical study was performed on 22 healthy Asian female volunteers older than 40 years. As a result, significant improvements in skin wrinkles for all of the five sites were observed after two weeks, as assessed by skin topographic measurements. Collectively, these results demonstrate the anti-aging efficacy of the peptides complex.

## 1. Introduction

The dermal-epidermal junction (DEJ) not only separates the epidermal and dermal compartments of the skin, but also provides a biochemical interface between the epidermis and the dermis. As a unique histological feature of the DEJ, a wavelike structure or undulation is known to be formed by both epidermal protrusions down into the dermis (rete ridges) and dermal projections up into the epidermis (dermal papillae). In intrinsically aged skin, the thinning and flattening of the DEJ structure is commonly observed, which results in a compromised adhesion of the epidermis onto the dermis and a consequent reduction of mechanical stability and structural integrity [1]. 

The interface area through which nutrients and signaling molecules move is also reduced. While a similar change was also observed for photoaged skin, additional structural impairment, expressed as a disrupted and multilayered lamina densa under an electron microscope, is also reported [2]. This impairment results from elevated expressions of extracellular matrix degrading enzymes, including matrix metalloproteinases (MMPs), urinary plasminogen-activator (uPA)/plasmin and heparinase, which can also degrade the epidermal basement membrane (BM) comprising proteins [3]. These structural changes and consequent diminution of molecular exchanges between the epidermis and the dermis result in various age-related changes, such as an impairment of epidermal permeability barrier functions [4], decreased wound healing [5] and skin dryness [6]. 

In addition to the structural impairment of the DEJ, age-related reductions of the basement membrane proteins comprising DEJ, including collagen I, collagen IV, collagen VII, collagen XVII, nidogen, integrin β4 and laminin-332, have been repeatedly reported [7,8]. Collagen VII and Collagen IV, which are produced by both keratinocytes and fibroblasts, are major components of anchoring fibrils, providing mechanical supports for the keratinocytes [9]. Collagen XVII is another structural component of the anchoring filaments, which play important roles in the assembly and functioning of the cell-matrix adhesion structure, as well as roles in transmembrane signal transduction and keratinocyte differentiation [10]. Laminins, as the most abundant glycoproteins of the basement membrane extracellular matrix, also play essential roles in the supporting the tissue’s architecture and stability [11]. Through the regulation of core cellular activities, such as adhesion, proliferation, migration, apoptosis and differentiation, laminins are involved in skin reepithelization and wound healing [12]. Along with perlecan, nidogen is assembled into the extracellular matrix as a bridging protein between collagen VI and laminin to form a basement membrane [13]. 

Based on the important roles of the DEJ in skin homeostasis, modulation of the basement membrane comprising proteins has been suggested as one of the potential strategies for improving skin wrinkles. Recently, the stimulation of collagen IV using a triple peptide complex, composed with a tripeptide (Arg-Gly-Ser), a hexapeptide ([Gly-Pro-Glc]_2_), and a dipeptide dimer ([H-Cys-Gly-OH)_2_), was reported as being effective for improving skin wrinkles [14]. In this study, we tried to develop new peptide derivatives with the potential anti-aging effects of reducing skin wrinkles, based on their BM protein modulating activities. As targets BM proteins, collagen XVII, laminin and nidogen were chosen on the basis of their epidermal origin. For this purpose, a series of peptide derivatives was tested based on prior investigations. Previously, the stimulation of collagen expression in human skin by growth hormone-releasing peptide-6 (GHRP-6) or its derivatives, and their cosmetic applications, were reported [15]. As a derivative of the growth hormone-releasing peptide (GHRP), we recently reported that biotin-conjugated GHRP-6 peptide (biotinyl-hexapeptide: BH) induced collagen and insulin-like growth factor-1 (IGF-1) expressions in cultured human myoblasts [16], which suggests a potential application for topical usage. 

The human cupper-binding peptide GHK-Cu is known to have multiple beneficial effects on human health, including skin rejuvenation. Along with supporting dermal fibroblast functions, this peptide also stimulates expressions of collagen, elastin and glycosaminoglycan, which makes it a widely used cosmeceutical peptide ingredient [17]. In order to improve the physical stability in its formulation and transcutaneous delivery profile, biotinyl-tripeptide (biotinyl-GHK: BT) was synthesized, and in this study, its effects on BM protein expressions were tested. Another collagen synthesis stimulating peptide, GEKG, developed by in silico screening [18], was selected for its potential activities on basement membrane protein expression. In order to increase the peptide’s biological activity, vitamin C was conjugated on the peptide using the succinyl group as a linker molecule. Considering the beneficial effects of vitamin C on the skin, stimulation of the proliferation and migration of dermal fibroblasts and the consequent stimulation of basement membrane re-organization [19], it was hypothesized that the conjugation of vitamin C with tetrapeptide GEKG (ascorbyl succinyl tetrapeptide: AST) can increase the expression of BM proteins. 

In this study, the effects of each of the peptides and peptides complexes were tested using in vitro cell culture models, and an ex vivo human skin model was used to explore the effects of the peptides complex on BM protein expression. The clinical efficacy of the peptide complex on facial wrinkles was measured to confirm the anti-aging effects of the peptides complex. 

## 2. Results

**Figure 1:**
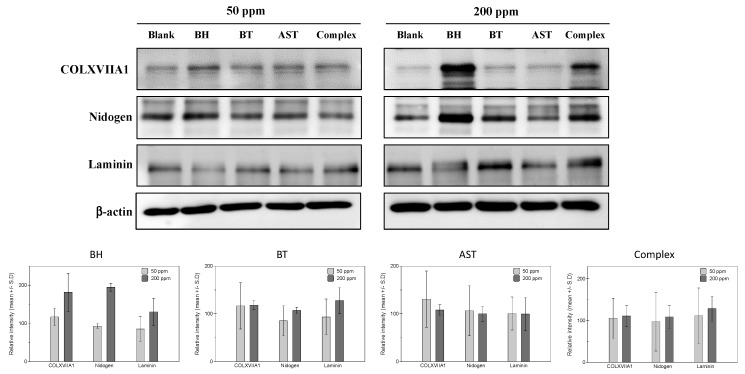
Effects of the test peptides on basement membrane protein expressions in human epidermal keratinocytes. At high concentrations (200 ppm), increased expression of COLXVIIA1 and nidogen was observed in biotinyl hexapeptide (BH)- and biotinyl tripeptide (BT)-treated cells. Laminin expression was stimulated by BT and BH treatment in high (200 ppm) concentrations, while a slight increase of laminin was observed in low (50 ppm) concentrated ascorbyl succinyl tetrapeptide (AST).

Based on the expression profiles in collagen, nidogen and laminin in human skin, cultured human epidermal keratinocytes and human dermal fibroblasts were adopted for in vitro efficacy measurement models. Firstly, protein expressions of collagen XVII, nidogen and laminin were measured in epidermal keratinocytes. From the preliminary experiments measuring the cytotoxicity of a tested peptide, two concentrations (50 ppm and 200 ppm) were decided for the further studies. As shown in Figure 1, both BH and biotinylated tripeptide (BT) resulted in increased expressions of collagen XVIIA1 protein, while only a slightly noticeable increase is observed in 200 ppm of ascorbyl succinyl tetrapeptide (AST)-treated cells. Similar changes in laminin and nidogen proteins expression were observed in BH- and BT-treated cells, and 200 ppm of BH and BT peptides treatment induced significant increase of both laminin and nidogen proteins’ expressions. Also, peptides complex treatment induced a noticeable increase of all the measured proteins at a 200 ppm concentration. These results suggest that the individual effects of each peptide on BM protein expression are also exerted as a mixture form.

**Figure 2:**
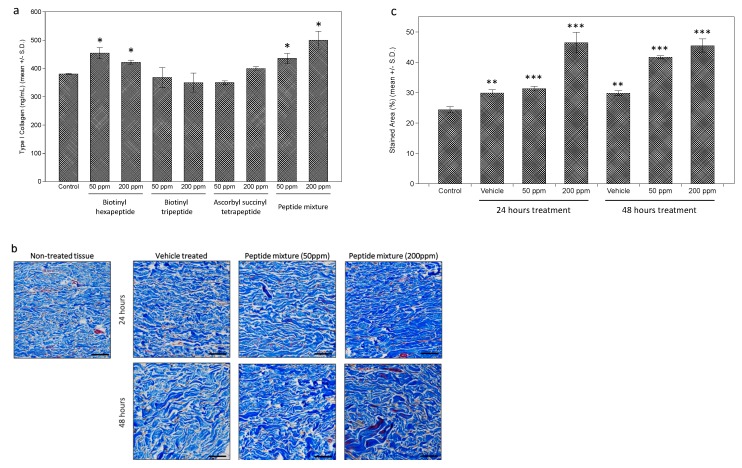
Stimulation of collagen expression in cultured dermal fibroblasts and ex vivo skin tissue. Increased expression of collagen I by tested peptides was observed in cultured human dermal fibroblasts by enzyme-linked immunosorbent assay (ELISA) (**a**). Ex vivo skin tissue treated with tested peptides were stained by Masson’s Trichrome staining (**b**), showing significantly increased intensity in peptides complex treated tissue (**c**). (bar = 100 mm). (*: *p* < 0.05; **: *p* < 0.01; ***: *p* < 0.001).

Further measurements of the effects on collagen expression were performed on the dermal fibroblasts. While each tested peptide and the peptides complex induced a slight increase in type I collagen expression, assessed by enzyme-linked immunosorbent assay (ELISA) analysis, the extent was as significant as expected (about a 1.3-fold increase in 200 ppm of peptides complex-treated cells) (Figure 2a). However, when collagen expression in dermal tissue was measured by Masson’s Trichrome staining after peptides complex treatment, a more significant increase was observed (Figure 2b). Through the image analysis using the ImageJ program, we calculated the stained area for each tissue, and about a 1.8-fold increase was observed in 200 ppm of the peptides complex-treated ex vivo tissue (Figure 2c). 

**Figure 3:**
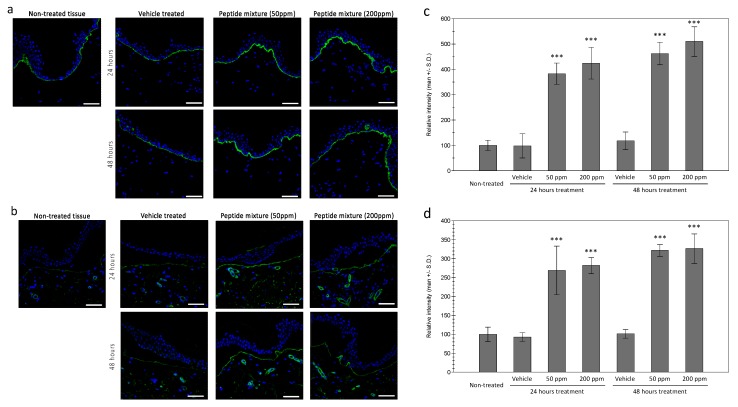
Expressions of collagen XVII and laminin in ex vivo skin tissue. Increased expressions of collagen XVII in the basal layer of the epidermis by tested peptide complex was observed by immunohistochemical staining (**a**). Dermal expression of laminin was also increased by peptide complex treated tissue (**b**). (bar = 100 mm). Fluorescence intensity analysis using ImageJ showed significant increases in both 24 and 48 h of treatment for collagen XVII (**c**) and laminin (**d**). (***: *p* < 0.001).

In order to confirm the expressions of collagen XVII and laminin in the treated tissue, immunohistochemical staining was performed in ex vivo tissue. As shown in Figure 3a, an increased expression of collagen XVII was observed in the epidermal basal layer in the treated tissue, compared to the vehicle treated tissue. Similar increases in laminin protein were observed in the peptides complex-treated tissue (Figure 3b). Interestingly, expression of the laminin protein was only observed in the uppermost layer of the dermal tissue and blood vessels. While the source of the laminin protein is epidermal keratinocytes, after expression, laminin is assembled with other basement membrane proteins and observed at the tips of dermal papillae, which is consistent with previous reports [20]. Similar increases of nidogen proteins in the uppermost layer of the dermal tissue were also observed.

**Figure 4:**
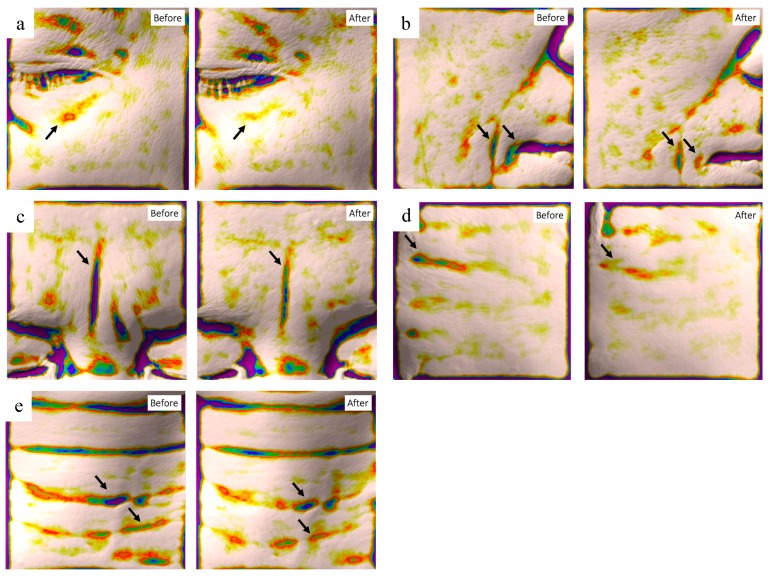
Reduction of skin wrinkles by peptide complex treatment. Representative images of Antera 3D photographs after image processing. Significant improvements of skin wrinkles (arrows) were observed in Crow’s feet (**a**), nasolabial folds (**b**), glabella frown lines (**c**), horizontal forehead lines (**d**) and horizonal neck lines (**e**).

To further evaluate the anti-aging efficacy of the peptide complex, a clinical study was performed on normal, healthy volunteers. A total of 22 female volunteers with a mean age (+/− standard deviation (SD)) of 52.4 (+/− 6.2) (min. 40; max. 60) completed the study, and changes in their face and neck wrinkles were assessed by topographic skin measurements using Antera 3D (Figure 4). After two weeks of usage, all of the measured wrinkles were significantly improved compared to the baseline value (Table 1). The highest improvement was observed for glabellar frown lines (12.51 +/− 7.86), while the lowest improvement was found for crow’s feet (6.09 +/− 7.38). 

## 3. Discussion

Among the various clinical features of aged skin, skin wrinkles are one of the most noticeable changes. While various kinds of causative factors, including chronic muscle contraction or gravitational forces, have been suggested to facilitate wrinkle formation, the quantitative decrease and qualitative deterioration of the extracellular matrix (ECM), which mainly supports the dermal layer, is considered to be one of the most critical factors [21]. Decreased expression of ECM proteins and increased activity of ECM degrading enzymes, including various matrix metalloproteinases (MMPs), as well as the structural impairment of filaments, are proposed to be underlying mechanisms for ECM impairment and have been major targets for conventional skin anti-aging strategies. Large numbers of studies have been published on the cosmetic ingredients for skin collagen synthesis stimulation, including ascorbic acid [22], retinol [23], natural extracts [24], growth factors [25] and collagen or its degraded forms [26]. Inhibition of ECM degradation, either through using MMP inhibitors, or down-regulating expressions of MMPs, is also suggested as an anti-aging regimen [27]. 

While commonly used, anti-aging ingredients generally target dermal fibroblasts and their functions, the recently identified importance of the dermal-epidermal junction (DEJ) on skin aging has suggested a new anti-aging mode of action focusing on epidermal keratinocytes. As a physical and biochemical interface between the epidermis and dermis, the DEJ acts as a connecting structure providing physical adherence and supporting molecular transport. At the DEJ, the basement membrane (BM) provides cell-adherent extracellular matrices, connecting the epidermis and dermis through its structural proteins. The core structural components of the BM are laminins, collagen IV, nidogens and heparin sulfate proteoglycans. Among these components, a decreased expression of collagen IV, laminin and nidogens in aged skin has been previously reported [7]. These proteins are initially secreted in a soluble state and then constitute complex insoluble scaffoldings, which provide cell and tissue support. BMs are also known to act as complex signaling platforms [13]. 

Among the major structural proteins comprising BM, the roles of collagen IV in skin aging were suggested by a series of in vitro and clinical studies. The reduction of collagen IV in aged skin and senescent fibroblasts was previously reported [28], and the stimulation of collagen IV expression in the skin was shown to improve skin wrinkles [14]. However, few studies have reported on the anti-wrinkle effects of laminin- or nidogen-stimulating compounds. Laminins belong to a family of heterotrimers consisting of α-, β- and γ-subunits, assembled through a long coiled-coil domain. While five α, four β and three γ chains are currently identified in mammals, only 16 different isoforms are observed in mammals. Recently, the important roles of laminin 522 during the differentiation of interfollicular stem cells and the establishment of proper structures in an in vitro 3D culture model were reported [20]. Also, the pentapeptide derived from the laminin b1 chain, tyrosine-isoleucine-glycine-serine-arginine (YIGSR), was shown to stimulate collagen synthesis in cultured human dermal fibroblasts [29], which also suggests the possible anti-aging effects of laminin-stimulating ingredients. Based on these results, and considering that many BM constituting proteins, including laminin and nidogen, are originally expressed in epidermal keratinocytes, secreted into the DEJ area, and assembled as BM protein complex [13], targeting epidermal keratinocytes seems a plausible approach for developing new anti-aging ingredients. In this study, we successfully identified that three different peptide derivatives having stimulatory activities on different members of BM proteins, and the peptides complex had anti-wrinkle activity ex vivo and in vivo. 

The cosmetic use of peptides as signaling molecules or carriers for metal ions is becoming more popular, mostly based on the identification of either new peptides sequences with beneficial biological activities [30], or new biological activities from previously used peptides [31]. In addition to the efforts in finding new peptides or new activities for known peptides, the derivation of peptides is also commonly used to develop new cosmetic ingredients [32]. While peptide derivation may discover novel activities, the common objectives of derivation are to increase the stability of peptide ingredients in the formulation and to improve bioavailability, mainly by increasing the transcutaneous delivery of peptides [33]. Biotinylation is one of the most commonly used derivation methods, and in this study, two kinds of biotinylated peptides were used for topical formulation. Consistent with the biological activities of original peptides, the simulation of BM protein expression was observed for each peptide derivative. Ascorbic acid conjugated tetrapeptide derivatives also exhibited postulated activities on nidogen expression, and the mixture of peptide derivatives showed the expected stimulating activities in all of the tested BM proteins, collagen XVII and laminin. 

As a result of a human clinical study to prove the anti-aging effect of the peptides complex, it was possible to observe the anti-wrinkle effect in various facial sites and the neck area after two weeks of usage. 

Previously, we reported that the topical application of a conditioned media of epidermal progenitor cells derived from mesenchymal stem cells, which contained high levels of nidogen-1, significantly improved various skin aging parameters, such as wrinkle and skin texture, which is consistent with current result [34] 

In this study, the potential application of epidermal protein expression-modulating ingredients as anti-aging cosmeceuticals was investigated. Three peptide derivatives with stimulatory activities on collagen XVII, laminin and nidogen in vitro, were used in a mixed form, and their anti-aging activities were examined using ex vivo and clinical studies. As a result, significant increases in dermal collagen, as well as epidermal protein expressions, were observed for peptides complex and further clinical studies also showed significant improvements in facial wrinkles. While this study suggests a possible correlation between BM proteins and dermal collagen expression, however, further investigations using siRNA or specific inhibitors are required for identifying a potential causality of BM proteins on extracellular matrix proteins expression. In conclusion, the current study suggests the anti-wrinkle benefits of the peptides complex, which stimulates expression of skin basement membrane proteins in vitro and ex vivo. These results also provide a new basis for exploring anti-aging molecules using a cultured keratinocyte and fibroblast model.

## 4. Materials and Methods

### 4.1. Materials

Among the tested peptides, biotinyl hexapeptide (BH) and biotinyl tripeptide (BT) were synthesized by the previously reported method [16]. Ascorbyl succinyl tetrapeptide-21 was supplied by CHA Meditech Ltd. (Daejeon, Korea). For preparation of the peptides mixture, equal amounts of each peptide were dissolved into the phosphate-buffered saline (PBS) solution, to make the final concentration of whole peptides as tested concentration, either 50 ppm or 200 ppm (*w*/*v*).

### 4.2. Cell Culture and Protein Measurement

The human neonatal primary dermal fibroblast (hDFn), human adult primary epidermal keratinocyte (HEKa) and culture media used for the study were purchased from Thermo Fisher Scientific (Waltham, MA, USA). hDFn was cultured with Medium 106 with a low serum growth supplement (LSGS) kit, and HEKa was maintained with EpiLife media with a human keratinocyte growth supplement (HKGS) and 1% antibiotics. The cells were cultured under 37 °C under a 5% CO_2_ condition. To measure protein expression, cells were lysed with a sample buffer (312.5 mM Tris-HCL (pH 6.8), 50% Glycerol, 5% SDS, 5% β-mercaptoethanol, 0.05% Bromophenol blue) and analyzed by Western blotting. After electrophoresis, the gel was blotted to a polyvinylidene difluoride (PVDF) membrane. the membranes were blocked for 1 h with 5% BSA in Tris-buffered saline with 0.05% Tween-20 (TBST) and then incubated with anti-Collagen XVII (rabbit, 1:1000 ab184996, abcam, Cambridge, UK), anti-Laminin (rabbit, 1: 1000 ab11575, abcam) and anti-Nidogen (rabbit, 1:1000 ab254325, abcam) antibodies overnight at 4 °C. The HRP conjugated secondary antibody was added to the primary antibody at room temperature for 1 h before washing the blots three times with TBST. The protein mass was compared after quantifying the intensity of the protein bands measured from three independent experiments by using a ChemiDoc (UVITEC, Cambridge, UK). To measure collagen type I expressions, hDFn cells were seeded in 12-well plates at a density of 5 × 10^4^ cells/well and incubated for 24 h, and then the cells were treated for 72 h with the samples. After 72 h, the supernatant was collected from each well, and the collagen type I contents were analyzed as follows. Goat Anti-Type I Collagen-UNLB (SouthernBiotech, Birmingham, AL, USA), diluted in a coating buffer (100 mM sodium bicarbonate buffer pH 9.6; Sigma, St. Louis, MO, USA), was added into a 96-well plate at 100 µL/well and coated overnight at 4 °C. After washing three times with PBS-T (0.05% tween-20 in PBS; Sigma), each well was blocked with a blocking buffer (0.5% BSA in PBS-T; genDEPOT, Katy, TX, USA) for 1 h. After removing the blocking buffer, the culture medium to be analyzed was added and allowed to react for 1 h. After washing three times with PBS-T, Goat Anti-Type I Collagen-BIOT (SouthernBiotech) was added and allowed to react for 1 h. 

After incubation with streptavidin conjugated horseradish peroxidase (Sigma) for 1 h, 3,3′,5,5′-tetramethylbenzidine (Sigma) was added and allowed to react for 20 min in the dark. Absorbance was measured with a Synergy HTX Multi-Mode Reader (BioTek Instruments Inc., Winooski, VT, USA) at a 450 nm wavelength.

### 4.3. Ex Vivo Skin Model

NativeSkin^®^ human skin models were obtained from Genoskin (Toulouse, France). The anonymized human skin sample used in this study was obtained from a female Caucasian donor aged 69 who underwent an abdominoplasty procedure and gave her written, informed consent. The donor did not have any record of allergies or dermatological disorders, and did not use corticosteroids. The collection, manufacture and use of skin models (NativeSkin) for research purposes were formally authorized by the French Ministry of Research (AC-2017-2897, 12 October 2017) and approved by the French Ethical Committee (Comité de Protection des Personnes (CPP)). Written informed consent was obtained from all participants. The study was conducted according to the Declaration of Helsinki protocols. Immediately following surgery, skin samples were processed. Subcutaneous adipose tissue was removed from the skin sample. Then, 11 mm diameter punch biopsies were excised and embedded in a proprietary biological matrix in transwells (Millicell) according to the patented NativeSkin^®^ procedure developed by Genoskin. The epidermal surfaces of the skin biopsies were left in contact with the air, and the dermal compartment was immersed in the matrix. The skin models were cultured up to 7 days in 12-well plates in a proprietary and chemically defined hydrocortisone-and-serum-free medium in the presence of 100 µg/mL penicillin and 100 µg/mL streptomycin in a humidified atmosphere of 5% CO_2_ at 37 °C. 

Upon arrival at the laboratories, skin models were acclimatized for 2 h in 12-well plates containing 1 mL of the maintenance medium (Genoskin) in a humidified incubator at 37 °C and a 5% CO_2_ condition. After 2 h, a fresh maintenance medium was added to the culture well and maintained for further experiments. Before the sample treatment, moisture on the skin surface was gently removed with a cotton tip, and 25 μL of the peptide complex solution (dissolved in an ethanol/PEG 400 (70/30) mixture) was applied directly on the stratum corneum. During the treatment period, skin models were placed in a humidified incubator at 37 °C and a 5% CO_2_ condition, with the maintenance medium changed every day. Each treatment was performed in duplicate.

### 4.4. Histology and Immunohistochemical Analysis

After the end of experiment, skin models were fixed in a 4% NBF (neutral buffered formalin) overnight at 4 °C and dehydrated in a graded ethanol series for paraffin embedding. Sections of 5 mm thickness were cut and mounted onto glass slides. The tissue sections were deparaffinized in xylene and rehydrated in a decreasing graded ethanol series before routine hematoxylin and eosin (H&E) staining. Trichrome staining for collagen expression observation was performed with a Masson’s Trichrome stain kit (HT15, Sigma-Aldrich, St. Louis, MO, USA). For quantitative analysis of the collagen expressions in the dermis, image analysis was performed using the ImageJ program. Each treatment was performed in duplicates and five fields were randomly taken and analyzed from one section, and a total of 10 fields were taken for image analysis for each sample.

For immunohistochemical staining, 5 mm-thick tissue sections were deparaffinized and rehydrated before heat-induced epitope revival treatment in an antigen retrieval solution (ab937, Abcam, Cambridge, MA, USA) at pH 6.0 for 10 min at 97 °C, followed by cooling for 10 min in a cooling chamber. After blocking the non-specific antibody binding using a blocking reagent (X0909, Dako), the rabbit anti-human collagen-17 antibody (ab184996, Abcam) or rabbit anti-human laminin antibody (ab11575, Abcam) were treated on the sections. The antibodies were diluted in an antibody diluent solution (S3022, Dako) and incubated in a humidified chamber overnight at 4 °C. The FITC-linked anti-rabbit antibody (ab15007, Abcam) was then applied to the tissue samples. The tissue sections were counterstained using a DAPI nuclear stain (#H-1200, Vector Laboratories, Inc., Burlingame, CA, USA).

### 4.5. Clinical Study

The clinical study (P1910-664) was performed on 22 Asian female subjects with facial wrinkles, aged from 40 to 60 years, who were approved by the Institutional Review Board of the P and K Skin Research Co., Ltd (1 October 2019). All studies complied with the World Medical Association’s Declaration of Helsinki (2013) concerning biomedical research involving human subjects. Female healthy volunteers without any skin or systemic diseases were initially enrolled for the study. Subjects who had retinoids or laser therapy within six months, or participated in other clinical study, were excluded. After hearing the explanation of the purpose and protocol of the study, all of the volunteers signed the informed consent and participated in the study. 23 female volunteers initially enrolled in the study, but during the study, one participant was dropped out due to the failure to present at the scheduled visit, and 22 participants completed the study. At the first visit, subjects were asked to complete study-related medical record questionnaires to confirm the inclusion and exclusion criteria, and each participant received written, informed consent. Before instrumental measurements, participants were asked to rest for at least 30 min in a humidity (40–60% RH) and temperature (20~25 °C) controlled room. Facial wrinkles in five areas (crow’s feet, nasolabial fold, glabella frown lines, horizontal forehead lines and horizontal neck lines) were measured by a topographic skin measurement device (Antera 3D CS: Miravex Ltd., Ireland). The participants were administrated to use test products containing 200 ppm of the peptide complex twice a day (morning and day) for two weeks. The participants were also asked neither to use any cosmetics containing ingredients that could potentially interfere with skin wrinkle status during the study period, nor to have any extensive exposure to direct sunlight. After two weeks of usage, 3D skin images were taken again, and image analysis was performed to calculate the change of their skin wrinkles. 

For the crow’ feet analysis, the saved images were transformed into a large wrinkle mode (10.7 mm diameter) and glabella frown line, forehead and neck lines were also analyzed using a large wrinkle mode (24.0 mm diameter). The maximum depth values within the circular analysis area were taken as the wrinkle-representing values, and the percentage reduction of those values before and after their application were calculated as the percentage improvement of skin wrinkles. For the nasolabial fold, the saved images were transformed into the large wrinkle mode at distinct areas, and the maximum depth values were measured [35]. 

### 4.6. Statistical Analysis

Parametric, two-tailed, paired *t*-tests, or non-parametric, Wilcoxon signed ranked tests were performed to compare the differences before and after application. The values are expressed as the arithmetic mean +/− standard deviation (SD). *p* values less than 0.05 were considered significant. 

## 5. Conclusions

In this study, peptides complex stimulating skin basement membrane proteins expression was developed and the anti-wrinkle benefits of the peptides complex was investigated in vitro and ex vivo. Clinical efficacy of peptide complex as anti-wrinkle cosmetic ingredient was also confirmed. 

## Figures and Tables

**Table 1:** The clinical efficacy of the peptide complex containing formulation. Significant improvement of facial and neck wrinkles assessed by image analysis using an Antera 3D camera (Miravex, Ltd.).

Measurement Sites	% Improvement (Mean +/− SD)	*p* Value
Crow’s feet	6.09 +/− 7.38	<0.001
Nasolabial fold	9.16 +/− 7.17	<0.001
Glabella frown lines	12.51 +/− 14.86	0.012
Horizontal forehead lines	10.64 +/− 7.86	<0.001
Horizontal neck line	8.67 +/− 7.83	<0.001
